# Perceived employment vulnerability in Latin America in the context of a potential new pandemic: a multicountry cross-sectional study

**DOI:** 10.1186/s12995-026-00521-9

**Published:** 2026-07-15

**Authors:** Christian R. Mejía, Víctor Serna-Alarcón, Jamil Cedillo-Balcázar, Leonel Vega-Useche, Dalia Useche-Villamizar, Camilo Vega-Useche

**Affiliations:** 1 Universidad de Huánuco, Huánuco, Peru; 2 Universidad Privada Atenor Orrego, Piura, Peru; 3 Hospital de la Amistad Perú Corea Santa Rosa II-2, Piura, Peru; 4 Estudiante de medicina, Carrera de Medicina, Facultad de Ciencias de la Salud, Universidad Técnica Particular de Loja, Loja, Ecuador; 5 Universidad Pedagógica y Tecnológica de Colombia, Tunja, Colombia; 6 Fundación Santa Fe de Bogotá, Bogotá, Colombia

**Keywords:** Occupational health, Employment vulnerability, Health emergencies, Social determinants of health, Latin America

## Abstract

**Introduction:**

Emerging infectious disease threats may reactivate concerns about employment instability and economic disruption, particularly in regions marked by labor informality, social inequality, and limited social protection. Although the effects of COVID-19 on employment have been widely documented, less is known about how workers perceive the potential labor consequences of future health emergencies. This study aimed to assess perceived labor-related repercussions of a potential new pandemic among workers in Latin America.

**Methods:**

An analytical cross-sectional study was conducted using secondary data from an online survey administered to workers from more than ten Latin American countries between June and July 2022, during the early phase of the 2022 mpox outbreak. Participants were asked about the expected impact of a potential new pandemic on their employment, salary, and overall work situation. Associations between perceived labor-related repercussions and sociodemographic and occupational factors were evaluated using Poisson generalized linear models with a log link and robust variance, adjusted for country of residence.

**Results:**

Among 850 surveyed workers, 64.2% perceived that a potential new pandemic would have a considerable or very strong impact on their work. In the adjusted analysis, men reported a lower perceived impact than women (aPR: 0.87; 95% CI: 0.78–0.97; *p* = 0.009). Lower perceived impact was also observed among those engaged in virtual work (aPR: 0.64; 95% CI: 0.44–0.93; *p* = 0.018) and hybrid work (aPR: 0.84; 95% CI: 0.73–0.97; *p* = 0.017), compared with in-person workers. No occupational sector showed a statistically significant difference compared with healthcare workers in the revised adjusted model, although the food sector had a higher, non-significant point estimate.

**Conclusions:**

Perceived labor-related vulnerability in the context of a potential new pandemic was substantial among workers in Latin America. Perceptions differed mainly according to sex and work modality, while occupational-sector estimates should be interpreted cautiously because of subgroup heterogeneity and limited precision.

**Supplementary Information:**

The online version contains supplementary material available at https://doi.org/10.1186/s12995-026-00521-9.

## Introduction

The COVID-19 pandemic had profound effects on labor markets, household economies, and workers’ well-being worldwide [1, 2]. In Latin America, these effects were especially relevant because the pandemic unfolded in a region characterized by labor informality, social inequality, fragile social protection systems, and marked occupational heterogeneity [3, 4]. Containment measures, mobility restrictions, and workplace closures disrupted employment conditions across multiple sectors, with particularly severe consequences for workers whose activities depended on in-person presence or unstable income sources [5]. These disruptions also affected household livelihoods and were associated with financial strain, psychosocial distress, and other adverse consequences for workers and their families [6–8].

During the early phase of the 2022 mpox outbreak, concerns emerged regarding the possibility that a new infectious disease emergency could reactivate restrictive public health measures and generate additional social and economic disruption [9, 10]. In that context, renewed uncertainty arose about employment continuity, salary stability, and the need to reorganize work through in-person, remote, or hybrid arrangements [11].

These concerns are particularly relevant for workers whose livelihoods depend on in-person activities, unstable income, informal labor arrangements, or limited social protection [12]. Although the employment consequences of COVID-19 have been documented in Latin America [13, 14], less is known about how workers in the region perceive the potential labor consequences of future health emergencies. This knowledge gap is relevant in a context of marked employment inequality, where occupational vulnerability may shape preparedness and resilience during crises. Therefore, the aim of this study was to assess perceived labor-related repercussions of a potential new pandemic among workers in Latin America and to examine their association with sociodemographic and occupational factors.

## Methods

### Study design and population

An analytical cross-sectional study was conducted using secondary data from an exploratory multicountry online survey administered to workers in Latin America between June and July 2022, during the early phase of the 2022 mpox outbreak in the region. Participants were recruited from Peru, Bolivia, Paraguay, Honduras, Colombia, Ecuador, Costa Rica, Mexico, Venezuela, Brazil, Chile, and other countries in the region represented in smaller numbers. The survey was conducted after ethics approval was obtained (Approval No. 0424-2022-UPAO), and no monetary or non-monetary compensation was provided for participation.

The study included adult workers who, at the time of the survey, were employed, resided in one of the participating countries, and provided informed consent to participate. Participants were recruited using non-probability snowball sampling. The initial survey link was distributed by the research team and country collaborators through multiple online channels, including professional and academic networks, work-related contact groups, email, messaging applications, and social media. Initial contacts were asked to share the survey with other eligible workers. No specific occupational sector was exclusively targeted; rather, the survey sought to include workers from diverse sectors and countries. Because of the exploratory nature of the study and the use of snowball recruitment, no formal probabilistic sample size target was established.

Before analysis, a quality-control process was performed. Records were reviewed for eligibility, informed consent, employment status, completeness of the variables required for the present analysis, and internal consistency. Of the 966 initial survey responses, 116 were excluded during this quality-control process because of incomplete information on work experience or perception-related variables. The final analytical sample included 850 workers.

## Variables and procedures

The survey was part of a broader questionnaire designed to assess social, occupational, and health-related perceptions during the early mpox outbreak. For the present secondary analysis, only variables related to employment, salary, perceived work-related repercussions, sociodemographic characteristics, and occupational conditions were used. The survey items used in the present secondary analysis are provided as Supplementary File 1, including the wording of the perception-related questions and their response options.

The main outcome variable was perceived labor-related impact in the context of a potential new pandemic. This was assessed through three perception-related items: expected changes in employment (it would increase, remain the same, decrease, or be lost), expected changes in salary (it would increase for most or for few, remain the same, or decrease for most or for few), and overall expected changes in work (no changes, few, moderate, considerable, or very many changes). For analytical purposes, the overall impact variable was dichotomized: the category of interest combined “considerable” and “very many” changes, while the comparison category combined “no changes,” “few,” and “moderate” changes. This recoded variable was used as the dependent variable in the bivariate and multivariable analyses. The employment-change and salary-change items were analyzed descriptively and were additionally explored in correlation analyses to assess whether the three perception-related items captured overlapping or distinct dimensions of perceived labor vulnerability.

Independent variables included sex (male or female), age (measured quantitatively in completed years), occupational sector (health, self-employment, education, public sector, food industry, mining, and other sectors), years of work experience, type of work (in-person, virtual, or hybrid), work modality (administrative or operational/field), and country of residence. For occupational sector, healthcare was selected as the reference category in the revised analyses to improve interpretability, given the heterogeneity of the “other sectors” category.

After ethics approval, data extraction was performed according to the predefined selection criteria. Data management and quality checks were conducted first in Microsoft Excel 2019 and then verified in Stata 18 before statistical analysis.

### Data analysis

Descriptive analyses were performed to summarize the sociodemographic, occupational, and perception-related characteristics of the study population. Frequencies and percentages were reported for categorical variables, whereas means and standard deviations, as well as medians and interquartile ranges, were reported for quantitative variables. Medians and interquartile ranges were prioritized for interpretation because quantitative variables showed non-normal distributions according to the Shapiro-Wilk test. The prevalence of perceived work-related impact was also described by country of residence, and between-country differences were explored descriptively using the chi-square test. Because several country-specific sample sizes were small, country-level findings were interpreted cautiously and were not considered primary exposure estimates. Country-specific estimates are presented in Supplementary Table 1**.**

Spearman correlation coefficients were estimated to explore the relationship among the three perception-related items: overall perceived work-related impact, expected employment change, and expected salary change. This analysis was performed to assess whether these items reflected related but non-equivalent dimensions of perceived labor vulnerability. The correlation matrix is presented in Supplementary Table 2**.**

Bivariate and multivariable analyses were conducted using generalized linear models from the Poisson family with a log link and robust variance, adjusted for country of residence. These analyses produced prevalence ratios, 95% confidence intervals (95% CI), and p-values. For bivariate models, each independent variable was evaluated in a separate country-adjusted model. In the multivariable model, variables associated with the outcome in the bivariate analyses were included together with country of residence. Healthcare was used as the reference category for occupational sector because it represented a defined occupational group and provided a more interpretable comparator than the heterogeneous “other sectors” category. Variables with a p-value < 0.05 in the bivariate analysis were entered into the multivariable model, and the same threshold was used to define statistical significance. All analyses were conducted in Stata version 18.

## Results

Among the 850 surveyed workers, Peru accounted for the highest proportion of respondents (26.9%). Women represented 54.1% of the sample. The median age of participants was 33 years (interquartile range: 27–42). The most frequently reported occupational sector was self-employment (25.1%). The median work experience was 10 years (interquartile range: 4–20). In-person work was the most prevalent arrangement (75.7%), and administrative work was the most frequently reported modality (58.9%) **(**Table 1**).**

**Table 1:** Sociodemographic and occupational characteristics of surveyed workers

Variables	Frequency	Percentage
Country		
Peru	229	26.9%
Bolivia	92	10.8%
Paraguay	81	9.5%
Honduras	79	9.3%
Colombia	37	4.4%
Ecuador	55	6.5%
Costa Rica	119	14.0%
Mexico	29	3.4%
Venezuela	35	4.1%
Brazil	43	5.1%
Chile	29	3.4%
Other	22	2.6%
Sex		
Female	460	54.1%
Male	390	45.9%
Age (years)*		
Mean and standard deviation	35.4	10.7
Median and interquartile range	33	27–42
Occupational sector		
Other sectors	140	16.5%
Health	204	24.0%
Self-employed	213	25.1%
Education	124	14.6%
Public sector	86	10.1%
Food industry	47	5.5%
Mining	36	4.2%
Work experience (years)*		
Mean and standard deviation	13.0	10.5
Median and interquartile range	10	4–20
Type of work		
In-person	643	75.7%
Virtual	41	4.8%
Hybrid	166	19.5%
Work modality		
Administrative	501	58.9%
Operational or field	349	41.1%

*Variable measured quantitatively

Regarding overall perceived impact, 37.7% of respondents believed that a potential new pandemic would have a very strong effect on their work, and 26.5% perceived a considerable effect, while 6.4% perceived no impact. Regarding employment, 6.8% anticipated job loss, whereas 38.8% expected a decrease. Regarding salary, the most frequent response was an expected decrease for most workers (43.9%), followed by no expected change (23.4%) and a decrease for only a few workers (21.6%) (Table 2).

**Table 2:** Perceived labor-related repercussions among workers in Latin America

Variables	Frequency	Percentage
Overall perceived impact		
None	54	6.4%
Few	93	10.9%
Moderate	157	18.5%
Considerable	225	26.5%
Very many	321	37.7%
Expected change in employment		
Increase	130	15.3%
Remain the same	332	39.1%
Decrease	330	38.8%
Be lost	58	6.8%
Expected change in salary		
Increase for most	12	1.4%
Increase for few	82	9.7%
Remain the same	199	23.4%
Decrease for few	184	21.6%
Decrease for most	373	43.9%

Perceived work-related impact varied by country, ranging from 39.5% in Brazil to 85.7% in Venezuela (χ² *p* = 0.002; Supplementary Table 1). These country-specific estimates were interpreted descriptively because several countries had small sample sizes.

The three perception-related items were statistically correlated, although the magnitude of the correlations was low to moderate (Supplementary Table 2)**.** Overall perceived work-related impact was weakly correlated with expected employment change (rho = 0.141; *p* < 0.001) and expected salary change (rho = 0.215; *p* < 0.001), while expected employment change and expected salary change showed a moderate correlation (rho = 0.365; *p* < 0.001).

In the country-adjusted bivariate analysis, perceived work-related impact was associated with sex (*p* = 0.007), virtual work (*p* = 0.010), and hybrid work (*p* = 0.010). No occupational sector showed a statistically significant difference compared with healthcare workers in the country-adjusted bivariate model **(**Table 3**).**

**Table 3:** Perceived labor-related repercussions in the context of a potential new pandemic, by sociodemographic and occupational factors in Latin America, 2022

Variables	Perceived work-related impact		PR (95% CI)	*p*-value
	No *n* (%)	Yes *n* (%)		
Sex				
Female	143 (31.1)	317 (68.9)	Reference category	
Male	161 (41.3)	229 (58.7)	0.87 (0.78–0.96)	0.007
Age (years)*	34 (27–43)	32 (27–41)	1.00 (0.99–1.00)	0.138
Occupational sector				
Health	66 (32.4)	138 (67.6)	Reference category	
Other sectors	57 (40.7)	83 (59.3)	0.91 (0.76–1.08)	0.276
Self-employed	89 (41.8)	124 (58.2)	0.89 (0.77–1.04)	0.147
Education	43 (34.7)	81 (65.3)	0.97 (0.82–1.15)	0.738
Public sector	26 (30.2)	60 (69.8)	1.09 (0.91–1.29)	0.351
Food industry	11 (23.4)	36 (76.6)	1.12 (0.92–1.37)	0.245
Mining	12 (33.3)	24 (66.7)	1.03 (0.79–1.34)	0.835
Work experience (years)*	11 (4–20)	10 (4–19)	1.00 (0.99–1.00)	0.198
Type of work				
In-person	208 (32.4)	435 (67.6)	Reference category	
Virtual	24 (58.5)	17 (41.5)	0.61 (0.42–0.89)	0.010
Hybrid	72 (43.4)	94 (56.6)	0.83 (0.72–0.96)	0.010
Work modality				
Administrative	190 (37.9)	311 (62.1)	Reference category	
Operational or field	114 (32.7)	235 (67.3)	1.07 (0.97–1.19)	0.170

*Variable measured quantitatively. Prevalence ratios (PR), 95% confidence intervals (95% CI), and p-values were obtained using generalized linear models from the Poisson family with a log link and robust variance, adjusted for country of residence

In the multivariable analysis, men reported a lower perceived impact (aPR: 0.87; 95% CI: 0.78–0.97; *p* = 0.009), as did individuals engaged in virtual work (aPR: 0.64; 95% CI: 0.44–0.93; *p* = 0.018) and those working in hybrid formats (aPR: 0.84; 95% CI: 0.73–0.97; *p* = 0.017). No occupational sector showed a statistically significant difference compared with healthcare workers in the revised adjusted model. Workers in the food sector showed a higher point estimate, but this association did not reach statistical significance (aPR: 1.18; 95% CI: 0.96–1.44; *p* = 0.108) **(**Table 4**).**

**Table 4:** Multivariable model of perceived labor-related repercussions in the context of a potential new pandemic, accounting for sociodemographic and occupational factors in Latin America, 2022

Variables	aPR	95% CI	*p*-value
Male sex	0.87	0.78–0.97	0.009
Occupational sector			
Health	Reference category		
Other sectors	0.98	0.82–1.17	0.823
Self-employed	0.95	0.81–1.10	0.476
Education	1.00	0.85–1.19	0.965
Public sector	1.11	0.93–1.32	0.239
Food industry	1.18	0.96–1.44	0.108
Mining	1.05	0.81–1.37	0.710
Type of work			
In-person	Reference category		
Virtual	0.64	0.44–0.93	0.018
Hybrid	0.84	0.73–0.97	0.017

Adjusted prevalence ratios (aPR), 95% confidence intervals (95% CI), and p-values were obtained using generalized linear models from the Poisson family with a log link and robust variance, adjusted for country of residence

## Discussion

In this multicountry exploratory study, perceived work-related vulnerability in the context of a potential new pandemic was substantial among surveyed workers in Latin America. Nearly two-thirds of participants reported that a new pandemic would have a considerable or very strong impact on their work. In the revised country-adjusted multivariable model, male sex and virtual or hybrid work arrangements were associated with lower perceived work-related impact. By contrast, no occupational sector showed a statistically significant difference compared with healthcare workers, although the food sector showed a higher point estimate that should be interpreted cautiously.

One of the main findings of this study was that workers engaged in virtual or hybrid modalities reported a lower perceived impact on their work. This may reflect the greater adaptability of these work arrangements during recent health emergencies, which allowed some workers to maintain job continuity and preserve work activities despite restrictions or workplace disruptions. Jobs involving administrative tasks, education, digital services, remote coordination, professional services, and other computer-based activities may be more easily adapted to virtual or hybrid formats than jobs requiring continuous physical presence, direct customer interaction, manual labor, or on-site production. Previous studies have shown that workers who were able to transition to remote or hybrid arrangements experienced lower levels of work disruption than those whose jobs required full in-person presence [15, 16]. This suggests that flexible work arrangements may function as a protective condition during public health crises.

However, remote work should not be interpreted as uniformly advantageous. Adapting to virtual work may involve challenges related to work-life balance, communication, collaboration, ergonomic conditions, and unequal access to digital resources [1, 15, 17]. In addition, the feasibility of virtual work is unevenly distributed across occupations and may itself reflect broader differences in labor stability, digital access, income security, and occupational privilege. Therefore, the lower perceived vulnerability observed among virtual and hybrid workers may not only reflect the modality itself, but also the structural advantages associated with occupations that can be performed remotely.

Workers in the food sector showed a higher point estimate of perceived impact, although this association did not reach statistical significance in the revised adjusted model using healthcare workers as the reference category. This finding should therefore be interpreted as descriptive and hypothesis-generating rather than confirmatory. Food-related occupations often require in-person participation, continuity of operations, direct interaction within production and distribution chains, and adherence to strict hygiene and safety standards during health emergencies. These characteristics may contribute to perceived vulnerability during pandemic scenarios. Previous research has emphasized the importance of preventive and control measures in food-related occupations, including personal protective equipment, enhanced hygiene practices, and staff training in infectious disease prevention [4–19].

Women reported a higher perceived impact than men, as reflected by the lower prevalence of perceived work-related impact among male participants in the adjusted model. This pattern may be related to the unequal distribution of unpaid domestic work, caregiving responsibilities, and role overload during health emergencies. Similarly, in Argentina, women experienced greater work-related and caregiving repercussions than men during the pandemic [12]. Prior research has also suggested that women may experience higher levels of stress and anxiety than men in crisis contexts, particularly when increased workload and supplementary responsibilities coexist [20]. These differences may be reinforced by traditional gender roles and social expectations, which can intensify pressure to balance paid work, domestic tasks, and caregiving.

The three perception-related items were statistically correlated, but the magnitude of the correlations was low to moderate. This suggests that expectations about overall work changes, employment continuity, and salary changes represent related but non-equivalent dimensions of perceived labor vulnerability. This distinction is important because workers may anticipate major changes in how work is organized without necessarily anticipating job loss or salary reduction. Conversely, concerns about salary or employment may occur even when the expected changes in work activities are less pronounced. For this reason, the overall perceived work-related impact item was retained as the primary outcome, while expected employment and salary changes were reported descriptively and explored through correlation analyses.

From a public health and occupational health perspective, these findings suggest that preparedness strategies for future health emergencies should incorporate labor vulnerability as a relevant dimension of social inequality. Workers are not equally positioned to absorb the labor consequences of new outbreaks or restrictive measures, and differences by sex and work modality may translate into unequal economic and psychosocial consequences. Policies aimed at labor continuity, income protection, occupational safety, and feasible flexible-work adaptation may therefore be particularly relevant in Latin American settings marked by informality, occupational heterogeneity, and limited social protection.

This study has several limitations. First, the use of non-probability snowball sampling limits external validity and prevents generalization to the broader Latin American workforce. Second, because the survey was administered online, selection bias is possible, particularly favoring participants with internet access, digital literacy, and connection to academic, professional, or work-related networks. Third, several country-specific and occupational subgroup sample sizes were small, which limited precision and prevented robust country-specific or sector-specific inference. Fourth, the cross-sectional design does not allow causal inference. Fifth, the outcome was based on self-reported perceptions rather than objectively measured labor changes. Sixth, the perception-related items were developed for an exploratory survey and were not part of a formally validated scale. Seventh, although the quality-control process excluded incomplete records, the available analytical dataset did not allow a detailed comparison of included and excluded respondents. Finally, residual confounding is possible because the survey did not include detailed measures of income, contract type, informality, household composition, caregiving burden, or previous direct employment disruption during COVID-19.

Despite these limitations, this study provides exploratory multicountry evidence on perceived employment vulnerability during the early phase of the 2022 mpox outbreak in Latin America. Future studies should address this topic using larger and more diverse regional samples, probabilistic or targeted sampling strategies where feasible, validated instruments, and longitudinal designs better suited to evaluating temporal relationships and actual labor outcomes during health emergencies.

## Conclusions

Perceived labor-related repercussions of a potential new pandemic were substantial among surveyed workers in Latin America. In the revised country-adjusted multivariable model, men and workers engaged in virtual or hybrid modalities reported lower perceived work-related impact than women and in-person workers, respectively. Occupational-sector estimates did not show statistically significant differences compared with healthcare workers and should be interpreted cautiously because of subgroup heterogeneity and limited precision. These findings highlight the relevance of sex, work modality, and broader occupational conditions in shaping perceived labor vulnerability during future public health emergencies.

## Supplementary Information

Below is the link to the electronic supplementary material.

Supplementary Material 1

Supplementary Material 2

Supplementary Material 3

## Data Availability

The dataset analyzed during the current study is not publicly available because it contains information that could compromise participant privacy, but it is available from the corresponding author on reasonable request.
