# Supplementary material for: Perceived employment vulnerability in Latin America in the context of a potential new pandemic: a multicountry cross-sectional study
Source: J Occup Med Toxicol. 2026 Jul 15;21:33. doi: 10.1186/s12995-026-00521-9 (PMC13656704; doi:10.1186/s12995-026-00521-9)
Supplement: Supplementary file 1 [file 12995_2026_521_MOESM1_ESM.docx]

**Supplementary File 1. Survey items used in the present secondary analysis**

This supplementary file presents the survey items used in the present secondary analysis. The original survey was part of a broader exploratory questionnaire designed to assess social, occupational, and health-related perceptions during the early phase of the 2022 mpox outbreak in Latin America. The items below correspond to the variables analyzed in this manuscript.

# Eligibility and sociodemographic variables

| **No.** | **Survey item / variable** | **Response options or format** |
| --- | --- | --- |
| 1 | Country of residence | Peru; Bolivia; Paraguay; Honduras; Colombia; Ecuador; Costa Rica; Mexico; Venezuela; Brazil; Chile; Other |
| 2 | Sex | Female; Male |
| 3 | Age | Completed years |

# Occupational variables

| **No.** | **Survey item / variable** | **Response options or format** |
| --- | --- | --- |
| 4 | Occupational sector | Health; self-employed; education; public sector; food industry; mining; other sectors |
| 5 | Years of work experience | Number of years |
| 6 | Type of work | In-person; virtual; hybrid |
| 7 | Work modality | Administrative; operational or field work |

# Perception-related variables

| **No.** | **Survey item / variable** | **Response options or format** |
| --- | --- | --- |
| 8 | Expected change in employment in the context of a potential new pandemic | Increase; remain the same; decrease; be lost |
| 9 | Expected change in salary in the context of a potential new pandemic | Increase for most workers; increase for few workers; remain the same; decrease for few workers; decrease for most workers |
| 10 | Overall perceived work-related impact in the context of a potential new pandemic | None; few; moderate; considerable; very many |

# Analytical coding used in the manuscript

For the main analysis, the item on overall perceived work-related impact was dichotomized. **“Considerable” and “very many”** were classified as perceived work-related impact, whereas **“none,” “few,” and “moderate”** were classified as no perceived work-related impact. The employment-change and salary-change items were analyzed descriptively and were explored using Spearman correlation coefficients.
