# Supplementary material for: Perceived employment vulnerability in Latin America in the context of a potential new pandemic: a multicountry cross-sectional study
Source: J Occup Med Toxicol. 2026 Jul 15;21:33. doi: 10.1186/s12995-026-00521-9 (PMC13656704; doi:10.1186/s12995-026-00521-9)
Supplement: Supplementary file 2 [file 12995_2026_521_MOESM2_ESM.docx]

| **Supplementary Table 1. Country-specific perceived work-related impact.** | | | |
| --- | --- | --- | --- |
| **Country** | **Total n** | **No impact n (%)** | **Perceived impact n (%)** |
| Other | 22 | 11 (50.0) | 11 (50.0) |
| Peru | 229 | 88 (38.4) | 141 (61.6) |
| Bolivia | 92 | 27 (29.3) | 65 (70.7) |
| Paraguay | 81 | 31 (38.3) | 50 (61.7) |
| Honduras | 79 | 22 (27.9) | 57 (72.2) |
| Colombia | 37 | 13 (35.1) | 24 (64.9) |
| Ecuador | 55 | 16 (29.1) | 39 (70.9) |
| Costa Rica | 119 | 39 (32.8) | 80 (67.2) |
| Mexico | 29 | 11 (37.9) | 18 (62.1) |
| Venezuela | 35 | 5 (14.3) | 30 (85.7) |
| Brazil | 43 | 26 (60.5) | 17 (39.5) |
| Chile | 29 | 15 (51.7) | 14 (48.3) |
| Total | 850 | 304 (35.8) | 546 (64.2) |
| Pearson chi-square test: χ²(11)=29.93; p=0.002. Country-specific estimates were interpreted descriptively because several countries had small sample sizes. | | | |
