# Supplementary material for: Perceived employment vulnerability in Latin America in the context of a potential new pandemic: a multicountry cross-sectional study
Source: J Occup Med Toxicol. 2026 Jul 15;21:33. doi: 10.1186/s12995-026-00521-9 (PMC13656704; doi:10.1186/s12995-026-00521-9)
Supplement: Supplementary file 3 [file 12995_2026_521_MOESM3_ESM.docx]

| **Supplementary Table 2. Spearman correlations among perception-related items.** | | | |
| --- | --- | --- | --- |
| **Variable** | **Overall perceived work-related impact** | **Expected employment change** | **Expected salary change** |
| Overall perceived work-related impact | 1.000 |  |  |
| Expected employment change | 0.141; p<0.001 | 1.000 |  |
| Expected salary change | 0.215; p<0.001 | 0.365; p<0.001 | 1.000 |
| Spearman correlation coefficients were used because the perception-related variables were ordinal. | | | |
